# Furin may contribute to proglucagon processing and glucagon-like Peptide-1 production in human alpha cells

**DOI:** 10.1016/j.molmet.2025.102259

**Published:** 2025-09-27

**Authors:** Janyne Koepke, Wentong Long, Amy Barr, Peter E. Light

**Affiliations:** Department of Pharmacology and the Alberta Diabetes Institute, Faculty of Medicine and Dentistry, University of Alberta, Edmonton, Alberta, T6G 2E1, Canada

**Keywords:** Proglucagon, GLP-1, Furin, PC1/3, Islet

## Abstract

**Objectives:**

While glucagon-like peptide-1 (GLP-1) production has been previously documented in human alpha cells, the steps regulating its production and secretion are poorly characterized. We investigated the enzymes implicated in proglucagon processing, characterizing their expression and localization in primary human alpha cells and αTC1/9 cells.

**Methods:**

Human alpha cells and αTC1/9 cells were maintained in control conditions or exposed to proinflammatory and Akt-activating stimuli to enhance GLP-1 levels. Proglucagon and convertase enzyme gene expression, protein content, and subcellular localization were evaluated by qPCR, Western Blot, and immunofluorescent microscopy, respectively.

**Results:**

Our data suggests that the canonical GLP-1-producing enzyme, Prohormone Convertase 1/3 (PC1/3), is poorly expressed and localized in alpha cells, while its homologue furin is optimally positioned for GLP-1 production. We also note that GLP-1 and glucagon processing occur in different subcellular compartments, creating two distinct pools of secretory granules which respond to similar secretory stimuli.

**Conclusion:**

Our study suggests that furin, rather than PC1/3, is positioned to process proglucagon into GLP-1, and despite coming from the same precursor molecule, GLP-1 and glucagon are separately packaged in primary human alpha cells.

## Highlights

• Human alpha cells produce GLP-1 and glucagon under native culture conditions.
• Furin is well-positioned for proglucagon processing in human alpha cells.
• GLP-1 and glucagon are packaged in separate secretory granules.
• Akt activation increases GLP-1 production and alters TGN trafficking dynamics.

## Graphical abstract

Graphical abstract created in BioRender. Koepke, J. (2025) https://BioRender.com/00jtutv.

**Figure:**
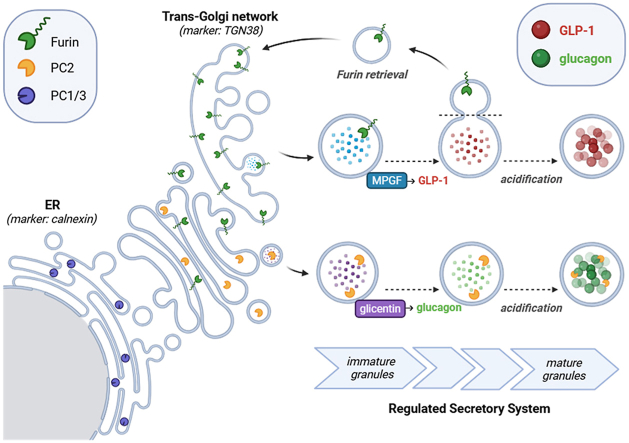

## 1 Introduction

Pancreatic islets are comprised of multiple endocrine cell types, including proglucagon-producing alpha cells, insulin-producing beta cells, and somatostatin-producing delta cells. Each cell type plays a unique role in the islet microenvironment, where coordinated paracrine and endocrine signalling mediates whole-body glucose homeostasis. Despite comprising up to 40% of human islet mass and exhibiting extensive functional heterogeneity [[1], [2], [3]], alpha cells have historically been overshadowed by beta cells in islet research [[4], [5], [6]]. Recent studies have highlighted significant alpha cell dysfunction in the etiology of Type 1 and Type 2 diabetes (T1D, T2D), contributing not only to beta cell dysfunction, but also to impaired intra-islet communication and systemic glucose regulation [2,[7], [8], [9], [10]].

Although alpha cells canonically synthesize and secrete the fasting hormone glucagon, several studies have documented their unexpected ability to produce the incretin hormone Glucagon-Like Peptide-1 (GLP-1) [3,[11], [12], [13], [14]]. Both glucagon and GLP-1 are derived from proglucagon, a multidomain precursor peptide, but its tissue-specific processing dictates which hormone is generated [15,16]. In alpha cells, proglucagon is processed by Prohormone Convertase 2 (PC2) to yield glucagon [17,18]. The emergence of alpha cell GLP-1, therefore, implies an alternative proglucagon processing event requiring an additional peptidase. In intestinal L-cells, Prohormone Convertase 1/3 (PC1/3) processes proglucagon into GLP-1 and GLP-2 [19], leading many to speculate that alpha cell PC1/3 production precedes intra-islet GLP-1 production.

Proglucagon-derived peptides are vital in coordinating beta cells’ glucose-responsiveness. Gαs-coupled receptors on beta cells, like the glucagon and glucagon-like peptide-1 receptors (GCGR, GLP1R), elevate cAMP to promote first-phase glucose-dependent insulin secretion [[20], [21], [22]]. Experimental evidence supports an intra-islet incretin axis, where beta cell-specific GLP1R knockout or administration of a GLP1R antagonist in isolated islets blunts glucose-dependent insulin secretion [23]. Furthermore, beta cell GLP1R expression is polarized such that receptors are concentrated on cell membranes in closest proximity to neighbouring alpha cells [24]. GLP1R agonists like semaglutide and liraglutide leverage these signalling pathways to improve insulin kinetics and whole-body glucose homeostasis in T2D management [25]. GLP1R blockade reduces insulin secretion in islets from T2D organ donors to a greater extent than healthy donors, suggesting a more pronounced role for beta cell GLP1R signalling in metabolic disease [26].

The existence of islet-derived GLP-1 raises important questions about its processing, packaging, and regulated secretion. While alpha cell PC1/3 could explain this shift in proglucagon processing, this mechanism remains unresolved and debated [3,[11], [12], [13], [14]]. Both ectopic PC1/3 production and PC2 knockdown enhance alpha cell GLP-1 production [[27], [28], [29]], but such manipulations do not accurately reflect human alpha cell physiology. Histological studies of human pancreatic slices have reported elevated PC1/3 in alpha cells from T1D and T2D organ donors [30,31]. Despite these studies demonstrating plasticity in alpha cell prohormone convertase expression, they did not explore the coexpression of GLP-1 and PC1/3.

Currently, there is no direct evidence linking PC1/3 to GLP-1 production in human alpha cells *in situ*, as recent histological investigations have disagreed on the prevalence of PC1/3 in GLP-1-positive alpha cells [31,32]. Another prohormone convertase family member, furin, was previously documented in human alpha cells [33]. Although recombinant studies demonstrate the ability of furin to cleave proglucagon to GLP-1 [34], its endogenous role in human alpha cells has not been characterized. This study is the first to comprehensively investigate alpha cell GLP-1, from enzymatic processing to regulated secretion, in primary human alpha cells. We characterize the proglucagon processing environment by assessing the abundance, maturation state, and subcellular localization of convertase enzymes, and examine the impact of cytokine and nutrient stimuli on GLP-1 production and release. Our findings suggest that furin, rather than PC1/3, contributes to proglucagon-to-GLP-1 processing in alpha cells, providing unique insight into islet-incretin biology.

## 2 Results

### 2.1 Alpha cells express glucagon, GLP-1, and multiple prohormone convertases

**Figure 1:**
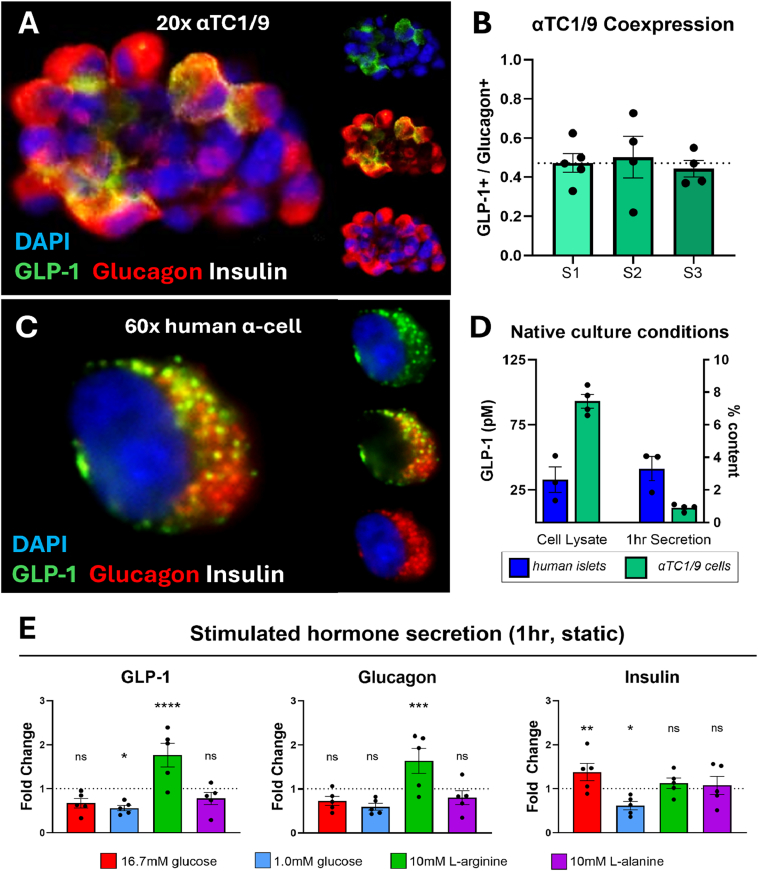
αTC1/9 cells and human islets express and secrete glucagon and GLP-1 under native cell culture conditions. A) Representative image of αTC1/9 cells counterstained with GLP-1 amide (green), glucagon (red), insulin (white) and DAPI nuclear stain (blue) at 20x magnification. B) GLP-1 amide and glucagon coexpression in αTC1/9 cells; 4–5 clusters analyzed per unique sample of αTC1/9 cells. C) Representative image of a single human alpha cell from donor H2572 counterstained with GLP-1 amide (green), glucagon (red) and DAPI nuclear stain (blue) at 60x magnification. D) GLP-1 [total] content normalized to 100 μg total protein (αTC1/9 cells) or 1000μIU/mL insulin (human islet lysate); N = 4 passages of αTC1/9 cells or 3 human islet donors. E) Static hormone secretion assays expressed as fold change from 5.5 mM glucose KRBH (basal) secretion conditions; ordinary two-way ANOVA with Dunnett's multiple comparison; Asterisks denote statistical significance (∗p < 0.05, ∗∗p < 0.01, ∗∗∗p < 0.001, ∗∗∗∗p < 0.0001); N = 4–5 islet donors.

We conducted parallel studies in intact human islets and αTC1/9 cells (a mouse-derived alpha-like cell line) to assess each model's intrinsic ability to produce glucagon and GLP-1. Like our previous findings in human alpha cells [3], only a subpopulation of αTC1/9 cells was GLP-1 positive, with all GLP-1 expressing cells staining glucagon-positive (Figure 1 A, B). We also confirmed that GLP-1 expression was not associated with a bi-hormonal insulin-expressing phenotype, as neither human alpha cells nor αTC1/9 cells expressing GLP-1 were insulin-positive (Figure 1 A, C). Next, we chose to compare each model's ability to produce and secrete GLP-1 as measured by GLP-1(total) ELISA in complete cell culture medium. When normalized to 1000uIU/mL total insulin, human islets contain approximately 100pM GLP-1(total), while αTC1/9 cells express approximately 100pM GLP-1(total) per 100ug tissue lysate (Figure 1D).

Both models release less than 5% of their GLP-1(total) content per hour under native culture conditions (Figure 1D), suggesting some degree of regulated rather than constitutive GLP-1 secretion. To assess this, we conducted static hormone secretion assays with intact human islets in basal and stimulating conditions. Expectedly, insulin secretion was potentiated in high glucose, suppressed in low glucose, and did not respond appreciably to amino acid stimulation in euglycemia (Figure 1E). GLP-1 and glucagon were released in response to 10 mM l-Arginine at 5.5 mM glucose, while 1.0 mM and 16.7 mM glucose suppressed the secretion of both hormones. We noted a near-identical fold-change in GLP-1 and glucagon secretion (Figure 1E).

**Figure 2:**
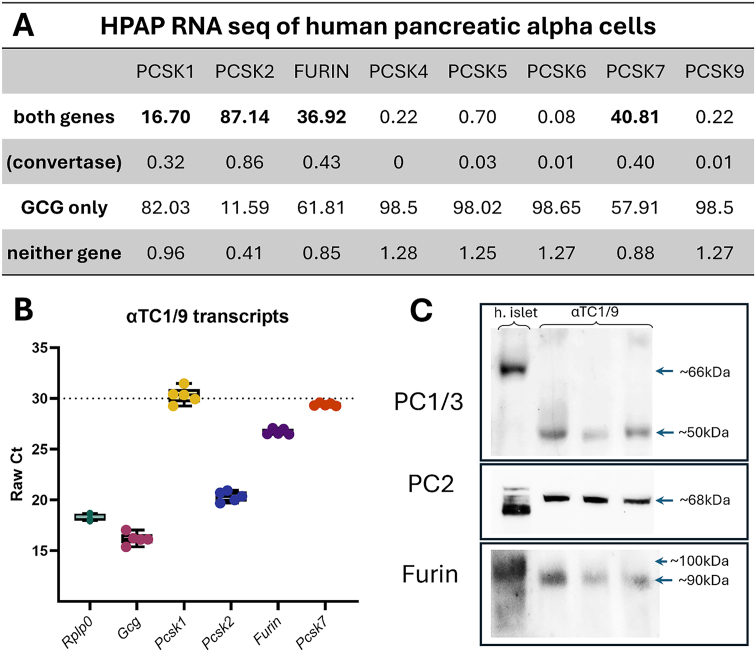
αTC1/9 cells and human islet serine proteases transcriptional and protein expression. A) Summary table of single cell RNA coexpression in human alpha cells from the Elgamal et al., 2023 HPAP dataset, generated using the ShinyCell web application available through https://www.gaultonlab.org/islet-genomics/ (accessed 04-27-2024). B) Raw Ct values from N = 5 separate passages of αTC1/9 cells conducted in biological and technical duplicates collected via SybrGreen qPCR. C) Representative blots of human islet lysate (donor H2587) and N = 3 separate passages of αTC1/9 cells; 40ug protein per lane; 1:200 PC1/3, 1:500 PC2, and 1:200 Furin primary antibody overnight on PVDF membrane.

Next, we aimed to identify which prohormone convertase enzymes were expressed in alpha cells and assess their roles in hormone processing. We utilized a single-cell HPAP (Human Pancreas Analysis Program) dataset to screen alpha cells for transcriptional coexpression of proglucagon (*GCG*) and different convertase enzymes. This analysis revealed *PCSK1, PCSK2, FURIN,* and *PCSK7* as transcripts of interest, which were co-expressed with *GCG* in a significant number of human alpha cells (Figure 2A). To determine the transcript abundance of each convertase enzyme, we next assayed αTC1/9 cells by qPCR. All four transcripts were detectable at varying levels in αTC1/9 cells, with transcript abundance consistent over five subsequent passages. *Pcsk2* and *Furin* were the most abundant convertase transcripts, while *Pcsk1* and *Pcsk7* fell near the assay's lower detection limit (Figure 2B). Due to its low transcriptional abundance and a literature-reported functional pH range incompatible with proglucagon processing in secretory compartments [35], chose to exclude *Pcsk7* (PC7) from further analysis.

We conducted immunoblots on human islet lysates and αTC1/9 to assess their prohormone convertase content and maturation state. PC1/3, PC2 and furin were detectable in human islet lysates at varying levels of pro-peptide and mature peptide forms (Figure 2C, lane 1). As intact islets contain several islet-endocrine cell types, we utilized αTC1/9 cells as a substitute for pure alpha cells. Again, all three convertase enzymes were detectable in αTC1/9 cells (Figure 2C, lanes 2–7). Despite producing significant quantities of GLP-1, PC1/3 was only present at 50 kDa, with no active 66 kDa PC1/3 detected. We found αTC1/9 PC2 exclusively in its 68 kDa active form and a furin band at 90 kDa (Figure 2C, lanes 2–7). Both bands are consistent with the mature, enzymatically active form of each enzyme. Overall, our immunoblots suggest that PC2 and furin can reach functional maturity, whereas PC1/3 does not achieve its active 66 kDa form in αTC1/9 cells.

### 2.2 Alpha cell prohormone convertases are arranged in distinct subcellular compartments

**Figure 3:**
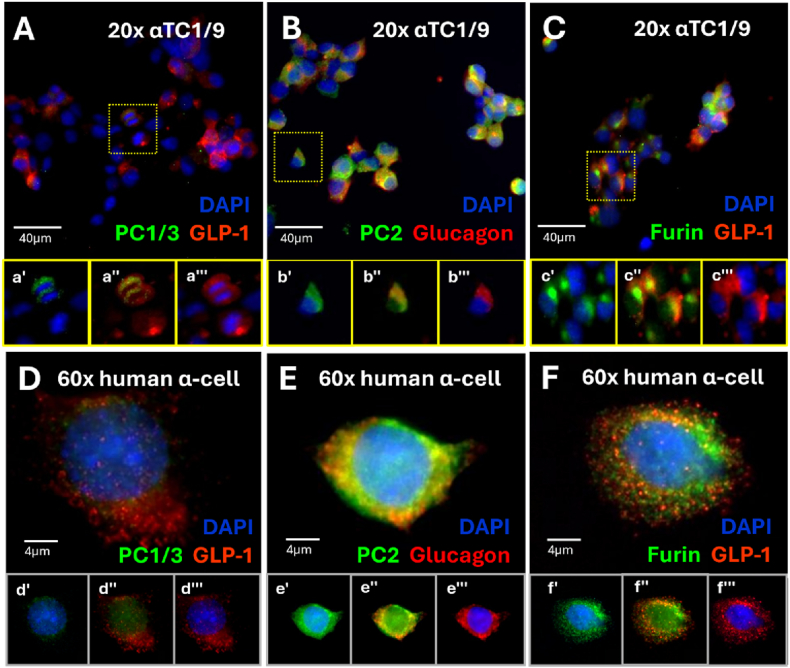
Histological analysis of glucagon, GLP-1, and their respective convertases in αTC1/9 cells and dispersed human islets. Brightfield immunofluorescent images of αTC1/9 cells (A–C) or dispersed human alpha cells (D–F). A and D are stained with 1:100 PC1/3 and 1:2000 GLP-1(amide), B and E are stained with 1:500 PC2 and 1:1000 glucagon, and C and F are stained with 1:100 furin and 1:2000 GLP-1(amide). Representative human alpha cell images were acquired from donor H2601.

Having confirmed transcript and protein expression with bulk techniques, we next aimed to characterize the coexpression of each convertase enzyme with either GLP-1 or glucagon. In our hands, only a subpopulation of αTC1/9 cells expressed PC1/3 (Figure 3A). Intriguingly, only half of GLP-1 expressing αTC1/9 cells stained PC1/3-positive, and these cells exhibited peri-nuclear localization of PC1/3. In contrast, PC2 and glucagon were co-expressed in all αTC1/9 cells (Figure 3B). Furthermore, all GLP-1 expressing αTC1/9 cells also expressed furin (Figure 3C). Similar to the αTC1/9 cells, PC1/3 was poorly detectable in human alpha cells with distinct perinuclear staining (Figure 3D), while PC2 and furin were expressed in all glucagon and GLP-1 positive cells, respectively (Figure 3E,F). All three convertase enzymes were abundant in human beta cells, with beta cell PC1/3 considerably more abundant than alpha cell PC1/3 (Figs. S1–S3).

**Figure 4:**
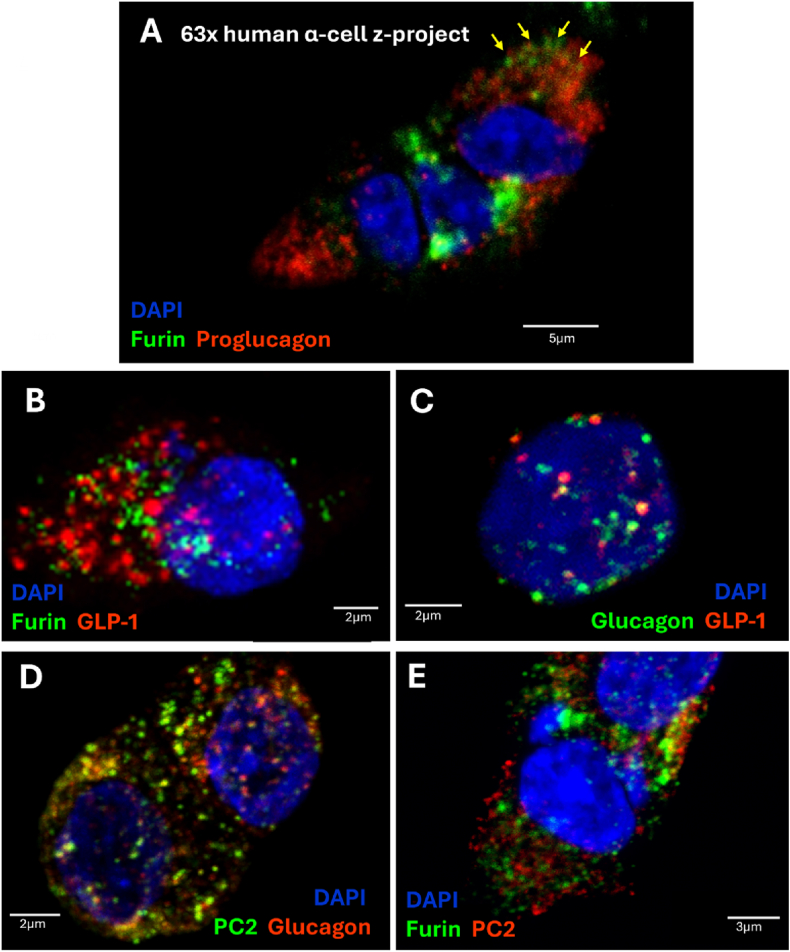
Confocal 63x z-projections of human alpha cells stained for proglucagon-derived peptides, furin, and PC2. A) stained with 1:100 furin and 1:1000 proglucagon. B) stained with 1:1000 glucagon and 1:2000 GLP-1(amide). C) stained with 1:100 furin and 1:2000 GLP-1(amide). D) stained with 1:500 PC2 and 1:1000 glucagon. E) stained with 1:100 furin and 1:500 PC2.

To investigate the subcellular localization of proglucagon-derived peptides and their relative convertases, we acquired confocal images of primary human alpha cells. At the subcellular level, furin colocalizes with proglucagon (Figure 4A) but not amidated GLP-1 (Figure 4B). GLP-1 and furin were localized in distinct compartments from one another, where furin vesicles were visually smaller than GLP-1 granules (Figure 4B). This staining profile suggested the selective sorting of furin from secretory granules into smaller compartments, consistent with furin's role as a trans-Golgi network (TGN) resident protein. We also found that glucagon and amidated GLP-1 are arranged in separate intracellular compartments with minimal evidence of co-packaging (Figure 4C). Further analysis confirmed that glucagon always colocalized with PC2, while PC2 was found in glucagon-positive and negative compartments (Figure 4D). Finally, we identified furin and PC2 as distinctly localized, with minimal overlap outside Golgi compartments (Figure 4E). Due to the cross-reactivity of antibody host species, we could not assay glucagon and furin, or PC2 and GLP-1 localization in the same samples.

**Figure 5:**
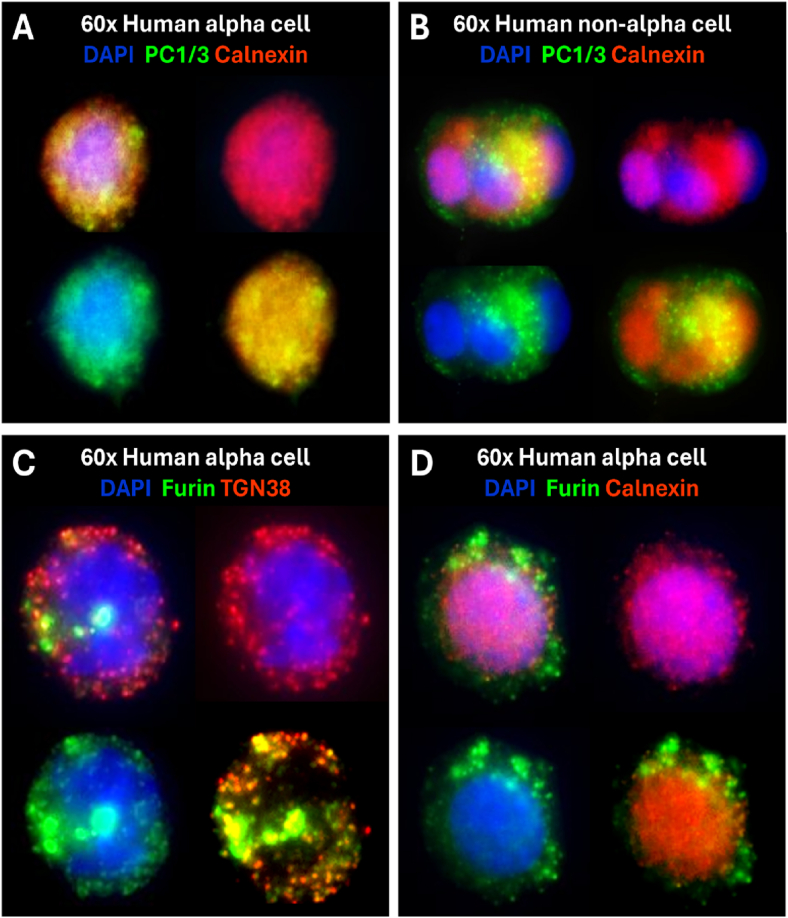
Subcellular localization of PC1/3 and furin in dispersed human islets. A) Representative image of a human alpha cell (proglucagon positive) stained with 1:100 PC1/3 and 1:500 calnexin. B) Representative image of a human non-alpha cells (proglucagon negative) stained with 1:100 PC1/3 and 1:500 calnexin. C) Representative image of a human alpha cell (proglucagon positive) stained with 1:100 Furin and 1:1000 TGN38. D) Representative image of a human alpha cell (proglucagon positive) stained with 1:100 Furin and 1:500 calnexin.

To determine the specific organellar localization of PC1/3 and furin, we stained dispersed human islet samples with the ER marker calnexin and the trans-Golgi marker TGN38. In human alpha cells, PC1/3 colocalized with the ER marker calnexin, indicating ER-retention (Figure 5A). Proglucagon negative cells exhibited PC1/3 staining in the periphery of the cell, confirming that PC1/3 can escape the ER in beta or delta cells (Figure 5B). Furin staining primarily overlaps with TGN38 (Figure 5C), with some calnexin colocalization (Figure 5D), indicating that most alpha cell furin is found in the TGN.

### 2.3 Furin-mediated GLP-1 production is feasible in alpha cells

**Figure 6:**
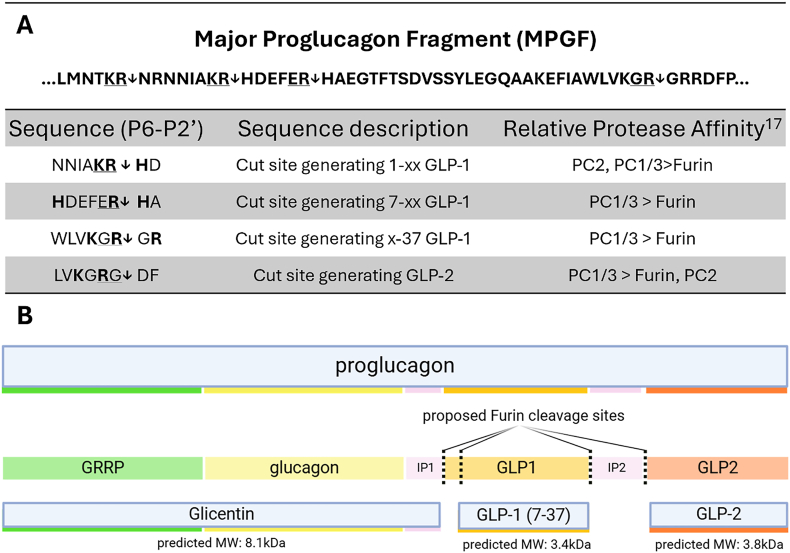
Analysis of furin's GLP-1 processing ability. A) Predicted Furin cleavage sites in the human Major Proglucagon Fragment sequence relevant to GLP-1 processing. Relative protease affinity was informed by Dey et al., 2005. B) Representative schematic of Furin-mediated proglucagon processing and its accompanying cleavage products*;* Created in BioRender. Koepke, J. (2025) https://BioRender.com/2y1s6df.

Furin's minimal cleavage consensus sequence is R-X-X-R, although basic residues like H, R, or K upstream of the cleavage site work to compensate for unfavourable residues in the consensus sequence [[36], [37], [38]]. Three major cleavage events are required to generate mature GLP-1 from the Major Proglucagon Fragment (MPGF). While none of these sites follow the furin minimal recognition sequence, all three have multiple basic residues (R, K, H) in the core P6–P2′ sequence that may compensate for energetically unfavourable binding interactions (Figure 6A). Our literature search identified previous publications characterizing the cleavage preferences of proglucagon by PC2, PC1/3, and furin *in vitro* [15,17,[39], [40], [41]]. These cleavage preferences are summarized in Figure 6B, predicting three major proglucagon-derived products: glicentin, GLP-1, and GLP-2 (Figure 6B).

### 2.4 GLP-1 is upregulated at the level of furin localization downstream of Akt activation

**Figure 7:**
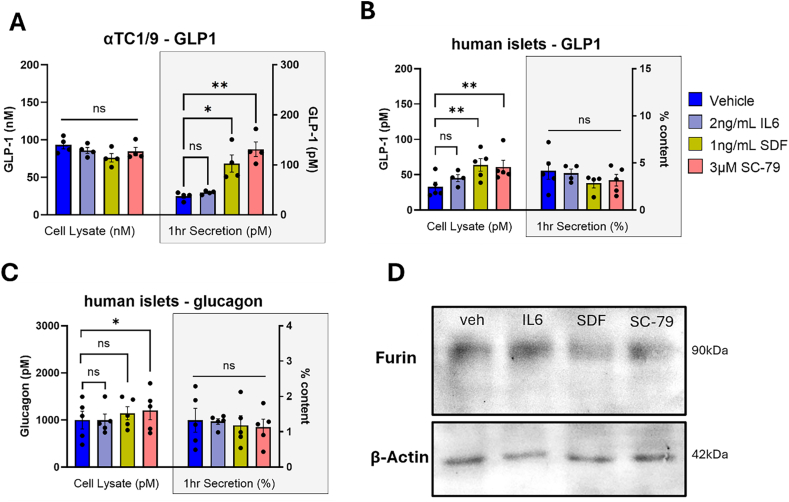
Proinflammatory cytokines and Akt activation increase alpha cell GLP-1 production but not furin levels. A) αTC1/9 cells assayed for GLP-1(total) and normalized to 100 μg total protein content, N = 4–5 passages. B and C) N = 5 human islet preparations assayed for GLP-1(total) (B) or glucagon (C). Human islet samples were normalized to 1000μIU/mL insulin content. D) Representative western blot of αTC1/9 lysate after 72 h incubation in vehicle conditions, 2 ng/mL IL6, 1 ng/mL SDF-1, or 3 μM SC-79. 20 μg whole cell lysate per lane, stained with 1:100 furin and 1:10,000 β-actin. Panels A–C: ordinary two-way ANOVA with Dunnett's multiple comparison; Asterisks denote statistical significance (∗p < 0.05, ∗∗p < 0.01, ∗∗∗p < 0.001, ∗∗∗∗p < 0.0001); N = 4–5 islet donors.

Our previous work suggested that cytokines IL-6 and SDF1α could induce GLP-1 expression in human islets [42]. Since both ligands are abundant in the T2D islet microenvironment, we assessed their effects on αTC1/9 cells and intact human islets over a 72-hour incubation assay. In contrast to previous studies [43,44], we employed physiologically relevant cytokine concentrations consistent with plasma levels in T2D. These were 2 ng/mL of IL6 and 1 ng/mL of SDF1α [45,46]. Since both cytokine receptor signalling pathways converge at Akt signalling, we also compared the direct effect of Akt activation by 3 μM SC-79, a small molecule that promotes Akt phosphorylation. After 72 h with each stimulus, αTC1/9 GLP-1 secretion, but not content, was significantly upregulated by SDF1α and SC-79 (Figure 7A). Parallel experiments in intact human islets had similar results, although GLP-1 content, not secretion, was upregulated by SDF1α and SC-79 (Figure 7B). Furthermore, we found no changes in human islet glucagon production with proinflammatory cytokines, and a modest but significant increase after 72 h of Akt activation by SC-79 (Figure 7C). Next, we assessed the relative abundance of furin and its maturation state after 72 h of drug treatment. αTC1/9 cells expressed appreciable amounts of furin in control conditions, and furin levels or molecular weight did not appreciably change after 72 h in IL6, SDF1α, or SC-79, despite their increased GLP-1 production (Figure 7D).

**Figure 8:**
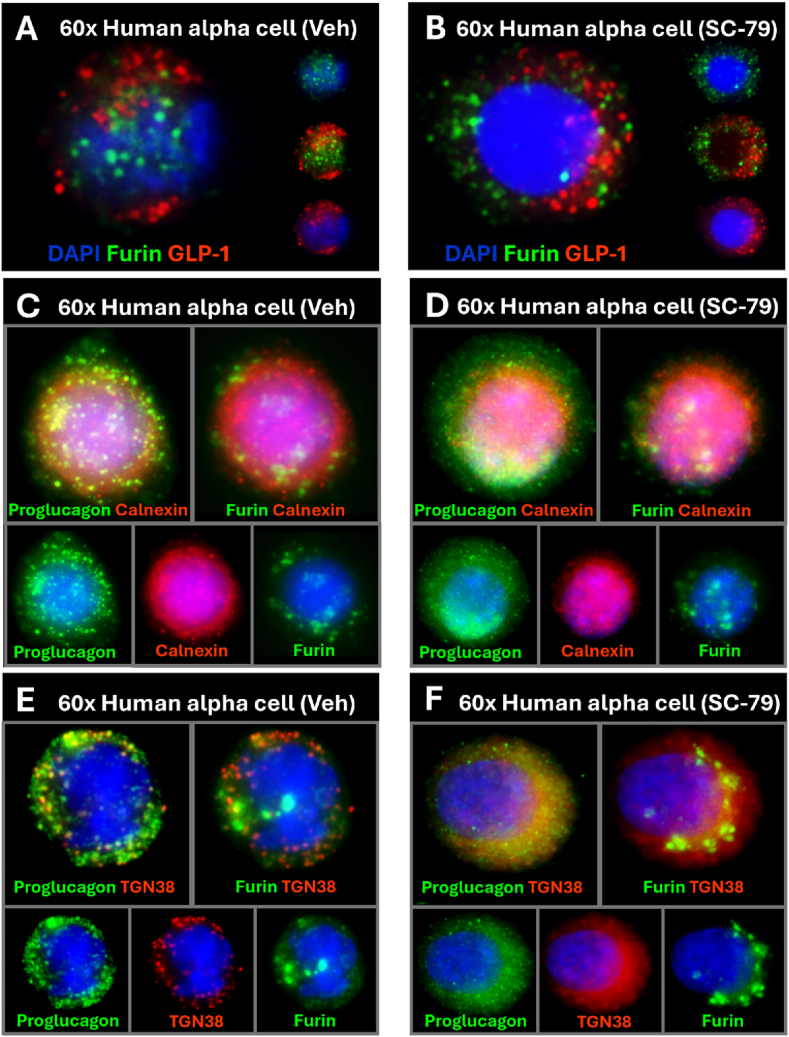
Akt activation alters human alpha cell TGN morphology. *Representative 60x max z-projections of human alpha cells.* A) Cultured in vehicle conditions for 72 h, stained with 1:100 furin and 1:2000 GLP-1(amide). B) Cultured in 3 μM SC-79 for 72 h, stained with 1:100 furin and 1:2000 GLP-1(amide). C) 4-channel image; cultured in vehicle conditions for 72 h, stained with DAPI (Ch1), 1:1000 proglucagon (Ch2), 1:500 calnexin (Ch3), 1:100 furin (Ch4). D) 4-channel image; cultured in 3 μM SC-79 for 72 h, stained with DAPI (Ch1), 1:1000 proglucagon (Ch2), 1:500 calnexin (Ch3), 1:100 furin (Ch4). E) 4-channel image; cultured in vehicle conditions for 72 h, with DAPI (Ch1), 1:1000 proglucagon (Ch2), 1:1000 TGN38 (Ch3), 1:100 furin (Ch4). F) 4-channel image; cultured in 3 μM SC-79 for 72 h, stained with DAPI (Ch1), 1:1000 proglucagon (Ch2), 1:1000 TGN38 (Ch3), 1:100 furin (Ch4).

To assess changes in furin localization, we imaged human islets treated with 3 μM SC-79. Vehicle-treated alpha cells maintained the expected ER/TGN localization staining pattern, while SC-79-treated cells exhibited more diffuse furin staining in the alpha cells' periphery (Figure 8A, B). To determine how furin localization was influenced by SC-79 treatment, we counterstained human alpha cells with proglucagon, furin, and an organellar marker. We noted some degree of proglucagon and furin colocalization with calnexin in vehicle and SC-79-treated cells (Figure 8C, D), but altered proglucagon granule morphology after SC-79 treatment (Figure 8D). TGN38 counterstains revealed proglucagon-containing compartments in the TGN and the cells’ periphery (Figure 8E), suggesting proglucagon is found in the Golgi and late secretory compartments of primary alpha cells. After SC-79 treatment, the degree of proglucagon-TGN38 colocalization increases, where proglucagon/TGN38 positive compartments appear punctate and smaller in apparent diameter after treatment (Figure 8F).

## 3 Discussion

### 3.1 GLP-1 production occurs independently of alpha-to-beta cell trans-differentiation

Our findings provide new mechanistic insights into GLP-1 production in human alpha cells. Here, we expand upon previous reports documenting GLP-1 production in alpha and alpha-like cells without experimental intervention, and delve into the mechanism of proglucagon processing, packaging, and secretion [3,12,[47], [48], [49]]. We observed a subpopulation of human alpha cells co-expressing glucagon and GLP-1, where the proportion of double-positive cells (∼40%) was consistent with past studies [3]. Similarly, 40–50% of αTC1/9 cells produced GLP-1, while all αTC1/9 cells stained glucagon positive. Our ability to upregulate GLP-1 production with inflammatory and Akt-activating stimuli, along with our previous observation of increased GLP-1-positive alpha cell area in T2D organ donors, suggests some degree of plasticity in alpha cell hormone processing phenotype [3]. Since our chosen methods did not allow us to temporally investigate the plasticity of alpha cell hormone processing, however, it is unclear whether all alpha cells can produce GLP-1, or only a distinct subset has this processing capability. Neither the GLP-1 positive primary cells nor αTC1/9s expressed insulin, confirming that this altered hormone processing profile was not the result of alpha cells adopting a beta cell-like phenotype. Finally, basal GLP-1 secretion from both models aligns with other peptides in the regulated secretory system —less than 5% per hour [50]. Overall, our findings suggest that intra-islet GLP-1 production is an alpha cell-intrinsic process, likely occurring in compartments of the regulated secretory system.

### 3.2 Endogenous PC1/3 expression does not account for alpha cell GLP-1 production

Although PC1/3 is widely understood to process GLP-1 in intestinal L-cells, our results do not support a similar role in alpha cells. Our study utilized the αTC1/9 clonal cell line, which is not expected to produce active 66 kDa PC1/3 and, by extension, GLP-1 [[47], [48], [49]]. In our hands, αTC1/9 *Pcsk1* transcripts fell near our assay lower detection limit, active 66 kDa PC1/3 was not detected by immunoblot, and PC1/3 immunofluorescent staining was weak. Despite this, αTC1/9s produced substantial levels of GLP-1, indicative of a PC1/3-independent processing pathway.

In primary human alpha cells, PC1/3 staining was weak, with peri-nuclear calnexin colocalization, indicative of ER retention. Several studies have confirmed that ER retention prevents PC1/3 maturation and compromises its ability to process substrates in the regulated secretory system [51,52]. Importantly, PC1/3 did not colocalize with amidated GLP-1 in mature secretory granules. Since it is not sorted from secretory granules before cargo condensation and secretion, PC1/3 is expected to colocalize with its processed substrates and adopt a similar colocalization profile to PC2 and glucagon [40]. The absence of PC1/3 from GLP-1 granules further supports the notion that PC1/3 does not contribute to proglucagon processing or GLP-1 production. While we detected 66 kDa PC1/3 in human islet lysate, this signal likely originated from beta cells, as only the 85 kDa autoinhibited form of PC1/3 is retained in the ER [[53], [54], [55], [56]]. This is supported by Figure S3, where PC1/3 is abundant in insulin-positive cells, with faint staining in GLP-1-positive cells. Furthermore, we noted that PC1/3 is poorly colocalized with calnexin in non-alpha cells and occupies peripheral (likely secretory) compartments, as seen in Figure 5B. These observations strongly suggest that PC1/3, when expressed in human alpha cells, cannot enter the regulated secretory system to meaningfully contribute to proglucagon processing or GLP-1 production. Our inability to detect 85 kDa PC1/3 in αTC1/9 lysate by immunoblot could stem from its subpopulation expression. We only observed PC1/3 peri-nuclear staining in half of GLP-1 positive cells, with only 40–50% of αTC1/9 expressing GLP-1. As such, we estimate PC1/3 expression in <20% of αTC1/9, likely falling at or below our assay detection limit.

### 3.3 Furin emerges as a plausible GLP-1 processing enzyme in alpha cells

We propose that furin, rather than PC1/3, may process GLP-1 in human alpha cells. Furin is a ubiquitously expressed peptidase with a documented but poorly characterized role in islet homeostasis. Previous studies have demonstrated that furin is required to process vacuolar proton pumps (V-ATPase) in alpha and beta cells, and its absence results in impaired proglucagon and proinsulin processing [57]. While this was attributed to impaired acidification of the secretory system, our data suggests that alpha cell furin may play a more direct role in proglucagon processing. Human pancreatic single-cell transcriptomic data revealed *FURIN* coexpression with *GCG* in approximately 37% of human alpha cells. In contrast, *PCSK1* was only detected in 16% of *GCG*-expressing alpha cells [58,59]. Our previous work identified GLP-1 expression in 35–40% of non-diabetic alpha cells, aligning with furin rather than PC1/3's transcriptional coexpression profile [3]. We confirmed appreciable *Furin* expression in αTC1/9 cells and detected protein bands at 90 kDa in αTC1/9 cells and human islet lysate, consistent with an N-terminally truncated active furin [60].

Furin was detected in all GLP-1-positive αTC1/9s and human alpha cells, and its colocalization with TGN38 confirms that furin can exit the ER. This staining pattern is significant since furin may only exit the ER after cleavage of its autoinhibitory N-terminus [61]. Despite their coexpression in alpha cells, furin and GLP-1 rarely colocalized at the subcellular level. Furin is uniquely excluded from mature secretory granules and only localizes with its cleavage products in transient compartments like immature secretory granules [[62], [63], [64]]. Due to the short half-life of immature secretory granules, colocalization of GLP-1 and furin is uncommon without experimentally stalling intracellular trafficking or furin's recycling process [63]. Instead, we observed subcellular colocalization of furin and proglucagon in human alpha cells, confirming that furin is well-positioned to process proglucagon into GLP-1, but could be sorted from secretory granules before cargo condensation. Furthermore, our analysis of intracellular compartments' diameter supports our hypothesis that furin is localized in clathrin-coated vesicles, a considerably smaller compartment than the mature secretory granule [62]. Together, these findings suggest furin localization is permissive of proglucagon processing in αTC1/9s and human alpha cells.

Although furin's minimal cleavage sequence (R-X-[K/R/X]-R) does not align well with proglucagon's GLP-1-generating cleavage sites, previously published studies demonstrate furin's ability to process proglucagon into glicentin, GLP-1, and GLP-2 [27,34]. The discordance between predicted and experimental furin activity may stem from furin's well-documented role in SARS-CoV-2 spike protein processing [37,65]. As this event occurs at extracellular pH conditions, most modern tools developed to predict furin-mediated substrate cleavage are trained on enzyme assays conducted at near-neutral pH conditions. In these pH conditions, basic residues within the cleavage consensus sequence would promote protein–protein interactions with furin, whereas acidic residues would lower the energetic favorability [36]. Acidic conditions, like in the regulated secretory system, can permit furin-mediated cleavage of substrates with lower isoelectric points. This is exemplified in hepatocytes, where proalbumin may only be cleaved by furin between pH 5.5–6, with no cleavage in extracellular pH conditions [66,67]. Dhanvatari et al. noted that only cells with a regulated secretory system (AT20 cells, GH3 cells) could produce GLP-1, whereas cells with a constitutive-only secretory pathway (BHK cells) could not [34]. Since furin's substrate recognition is highly dependent on peptide solubility and isoelectric point [36,37], proglucagon processing may be pH-dependent and unable to occur at neutral conditions like the ER, but more favourable in acidic compartments of the late-Golgi and regulated secretory system. Similar to proalbumin processing, proglucagon processing may appear energetically unfavourable to *in silico* tools trained in non-secretory pH conditions and restricted to acidic pH conditions *in situ.* Based on our histological observations, proglucagon-to-GLP-1 processing likely occurs in the late TGN or early secretory system, compartments ranging from pH 5.5–6 where furin is enzymatically active and substrate protonation is favourable to allow furin-mediated processing.

### 3.4 Akt signalling enhances GLP-1 production

Our previous investigation found increased islet GLP-1 expression in T2D, leading us to test the effect of the T2D-associated cytokines IL6 and SDF1α on proglucagon processing [3,[44], [45], [46],^68^,^69^]. While IL6 and SDF1α were already known to increase GLP-1 production in alpha cells, previous studies employed supra-physiological concentrations consistent with sepsis rather than metabolic inflammation [44,70,71]. We used mild cytokine concentrations and interrogated cell-signalling pathways downstream of cytokine receptor activation. From a pathological perspective, increases in islet IL-6 or SDF1α levels during metabolic inflammation and diabetes may lead to enhanced furin-mediated GLP-1 production [43,45,68]. This proposed mechanism is consistent with previous observations of increased intra-islet GLP-1 in patients with diabetes, and may be indicative of alpha cell remodelling to cope with metabolic stress [3,72]. Other researchers have postulated a greater reliance on islet-derived GLP-1 for supporting insulin dynamics, especially in individuals with low circulating GLP-1 [26].

Since both cytokine receptor signalling cascades converge at PI3K/Akt signalling, we treated our cells with a small molecule Akt activator, SC-79. We found that 2 ng/mL and 1 ng/mL of IL6 and SDF1α were sufficient to upregulate GLP-1 production in both models without compromising cell survival, and SC-79 recapitulated the effect of both cytokines. We suspect that Akt or one of its downstream effectors modulates alpha cell GLP-1 production, and other cell surface receptors converging at Akt signalling may have a similar up-regulatory effect. Such receptors could include other tyrosine-kinase-like receptors or Gq/11-containing GPCRs, including INSR, GPR120, or GPR142 [42,[73], [74], [75]]. This suggests that paracrine signals, nutrients, and metabolites may also contribute to alpha cells' hormonal phenotype in the absence of inflammation. Several investigations have noted that GLP-1 receptor signalling at beta cells can alter alpha cells’ hormonal phenotype, and secreted factors from beta cells can enhance alpha cell *Pcsk1* expression [[76], [77], [78]]. While none of these studies assay PC1/3 protein expression or colocalization, they establish beta cells as important mediators of alpha cell hormone processing phenotype. Alpha cell-specific GPCRs or surface receptors may represent a compelling therapeutic target where selective Akt activation could enhance intra-islet GLP-1 production before the onset of metabolic dysfunction. Understanding which receptors can selectively activate alpha cell Akt signalling may open the door to targeted therapeutic approaches that induce furin-mediated proglucagon processing and intra-islet GLP-1 production.

While furin's protein abundance or molecular weight did not change after Akt activation, changes in the morphology of proglucagon-containing and TGN38-positive compartments occurred. Furin is known to be recycled to the TGN via clathrin-coated vesicles, where its localization is modulated by phosphorylation state and unique adaptor protein complex assembly [62,[79], [80], [81]]. Future studies could explore the effects of Akt signalling on furin recycling at the level of casein kinase-II [81], protein phosphatase A2 [82], or adaptor protein assembly [62,83]. Furthermore, Akt-mediated phosphorylation of coat proteins and Rab GTPases is known to accelerate anterograde transport of COPII ER vesicles and secretory compartments [84,85]. Our observation of altered TGN morphology could be indicative of Akt-mediated changes in alpha cell intracellular trafficking, perhaps linking furin recycling and anterograde trafficking to favour proglucagon-to-GLP-1 processing.

### 3.5 GLP-1 and glucagon production occur in distinct intracellular compartments

Intriguingly, we noted that GLP-1 production did not occur at the expense of glucagon production, where SC-79 treatment increased GLP-1 and glucagon content in primary tissue. This data suggests that glucagon and GLP-1 processing may occur via parallel processing pathways. We propose that, rather than proglucagon being processed by either PC2 or furin, intermediate products glicentin and MPGF enter divergent processing pathways to yield glucagon and GLP-1, respectively. This hypothesis is supported by previous studies demonstrating that glicentin can be processed into mature glucagon by PC2, while the MPGF can be directly processed into GLP-1 [16,17].

Confocal microscopy supported our hypothesis that GLP-1 and glucagon processing take place in separate intracellular compartments. We observed minimal overlap between GLP-1 and glucagon-containing granules in primary human alpha cells. This was further supported by the distinct localization of each hormone's respective processing enzyme. Furin and PC2 have unique pH and calcium requirements, which correlate to distinct compartments within the TGN and regulated secretory system [39,86,87]. Without live cell imaging or pulse-chase style assays, however, we cannot conclude the intracellular compartments where each processing event takes place.

Despite their differential processing and packaging, we observed near-identical fold changes in glucagon and GLP-1 secretion in response to nutrient stimuli. These findings suggest that mature secretory granule pools may rely on the same exocytotic machinery, irrespective of cargo. Glucagon granule exocytosis is intrinsically linked to P/Q-type voltage-gated Ca2+ channel activity [88], yet recent electrophysiology studies note that some alpha cells have a preference for L-type Ca2+ channel-associated exocytosis [2]. It is possible that GLP-1-containing secretory granules are coupled to an alternative secretory pathway, like L-type Ca2+ channels, which were not selectively activated by our chosen stimuli. We only observed GLP-1 secretion in native-condition islets. Therefore, the effect of Akt-mediated GLP-1 upregulation must be further investigated to determine if increased GLP-1 production alters alpha cell secretion dynamics.

## 4 Study limitations

Although our study did not include enzyme activity assays, the proximity of furin to proglucagon and the presence of a lower molecular weight active furin protein band support the hypothesis that furin mediates GLP-1 production *in situ.* Despite these promising findings, functional validation is required to establish a direct role for furin in alpha cell proglucagon processing. Currently, no small-molecule furin-specific inhibitors exist. Due to shared sequence homology between convertase family members, existing inhibitors interact with all three of PC1/3, PC2 and furin. As such, reversible furin inhibition was not feasible in our model system. Future studies could employ siRNAs or CRISPR-Cas9 to eliminate alpha cell furin and establish the direct link between furin function and GLP-1 production. Previous PDX1-cre driven furin knockout mice studies identified impaired proinsulin and proglucagon processing [57]. Presumably, an alpha cell-specific furin knockout would compromise proglucagon processing at the level of granular pH and mask furin's direct role in GLP-1 production. Alternative approaches could target elements of furin's trafficking and recycling machinery to maintain appropriate V-ATPase assembly and subcellular pH while restricting furin's ability to exit the TGN and associate with proglucagon.

Our study did not utilize rodent islets or stem-derived alpha cells. We focused on primary human tissue due to species differences in islet architecture, alpha cell abundance, and translatability. For instance, we have previously documented a 50-fold lower GLP-1 secretory capacity in mouse islets than human islets, despite expressing ∼5-fold fewer alpha cells than human islets [3]. Our past investigations identified species differences in alpha cells’ capacity to produce and secrete GLP-1, requiring primary human alpha cells or a humanized alpha cell model for translatability. At this time, there are no optimized stem cell-derived alpha cell differentiation protocols that yield a population of “proglucagon-only” alpha cells. Instead, most stem-derived alpha-like cells harbour a functionally immature transcriptome with detectable *INS, IAPP,* and *SST* transcripts [89]. Additionally, these cells can transdifferentiate into other islet endocrine cells or exhibit compromised cell identity, something we did not observe in GLP-1-producing primary alpha cells. With existing differentiation protocols, a stem-derived alpha cell is not guaranteed to recapitulate the low-PC1/3, high-furin phenotype we observe in primary human alpha cells. Instead, these stem-derived cells may compensate for genetic manipulation (i.e. *FURIN* knockout) by altering their phenotype in a non-translatable manner. Continued refinement of stem-derivation protocols is essential to produce translatable, renewable alpha cell models. Until then, primary human islets remain the best available model for investigating alpha cell GLP-1 production.

Our reliance on cadaveric islet donations presents additional limitations, as the acquisition and isolation process can fundamentally alter their function [90]. We acknowledge that pancreas acquisition, islet digestion parameters, and *ex vivo* cell culture time contribute to islet health and function. A recent study by Kolic et al. noted a correlation between organ cold ischemic time and insulin secretory response, where extended pre-isolation time compromised beta cell function [91]. There is potential that islet-derived GLP-1 is negligible in healthy islets *in vivo* and, instead, the result of cadaveric tissue acquisition. We have previously confirmed that GLP-1 is produced in alpha cells from live donor biopsies, although these tissue donors had underlying pancreatitis and may not faithfully represent “normal” physiology [42]. Despite this, we are confident that islet GLP-1 production is not exclusively a result of cadaveric tissue isolation, and cytokine-mediated alpha cell GLP-1 production still represents a relevant mechanism for T2D islets, given the proinflammatory environment observed in metabolic disease [45,46,92,93]. While unlikely to contribute to systemic GLP-1 pools, we believe that alpha cell-derived GLP-1 has the potential for paracrine signalling on nearby endocrine tissue.

Peptidylglycine Amidating Monooxygenase (PAM), expressed in alpha and L-cells, is required to remove the C-terminal glycine residue from x-37 GLP-1 and amidating the resultant C-terminus [94,95]. Our confocal investigation of GLP-1 and glucagon-containing granules used a GLP-1(amide) antibody. While non-amidated GLP-1 represents <25% of total GLP-1 produced in L-cells [96], the proportion of amidated versus non-amidated GLP-1 levels has yet to be determined in alpha-cells. We cannot dismiss the possibility that non-amidated GLP-1 and mature glucagon may still be co-packaged while amidated GLP-1 is separately sorted. Furthermore, the MSD U-Plex total GLP-1 ELISA kit preferentially detects amidated species. Therefore, our GLP-1 measurements may underrepresent total GLP-1 (amidated and non-amidated) in our model systems. Currently, commercially available non-amidated GLP-1 antibodies lack specificity between MPGF and GLP-1, although analyzing human islet samples with multiple commercial antibodies may be beneficial in future studies.

## 5 Conclusion

Our study provides compelling evidence that proglucagon-to-GLP-1 processing in human alpha cells occurs independently of PC1/3 and may be mediated by furin. We demonstrate that active furin is present in GLP–1–producing alpha cells and localizes in a manner consistent with activity in the TGN and regulated secretory pathway. In contrast, PC1/3 is weakly expressed, retained in the ER, and absent from GLP–1–containing granules, rendering it unable to reach the regulated secretory system and unlikely to contribute meaningfully to proglucagon cleavage in this cell type.

Furthermore, we show that GLP-1 production is upregulated through physiologically relevant cytokine exposure and pharmacological Akt activation. Our findings suggest that TGN trafficking is influenced by Akt signalling to favour increased proglucagon-to-GLP-1 processing, although we did not establish a causative link between Akt activation and changes in furin trafficking or abundance. Finally, GLP-1 and glucagon appear to be processed in parallel but spatially distinct compartments, enabling alpha cells to simultaneously produce both peptides without compromising glucagon output.

These findings challenge the canonical view that PC1/3 is the universal GLP–1–producing enzyme and highlight the alpha cell's capacity to produce bioactive incretin under metabolic stress. Leveraging this local paracrine source of GLP-1 through targeted modulation of furin activity or alpha cell signalling may offer a novel strategy to support beta cell function in type 2 diabetes.

## CRediT authorship contribution statement

**Janyne Koepke:** Writing – review & editing, Writing – original draft, Visualization, Methodology, Investigation, Funding acquisition, Formal analysis, Data curation, Conceptualization. **Wentong Long:** Writing – review & editing, Resources, Methodology, Investigation. **Amy Barr:** Writing – review & editing, Supervision, Resources, Methodology, Investigation. **Peter E. Light:** Writing – review & editing, Supervision, Resources, Project administration, Funding acquisition, Conceptualization.

## Declaration of competing interest

The authors declare the following financial interests/personal relationships which may be considered as potential competing interests: Peter E. Light reports financial support and administrative support were provided by Canadian Institutes of Health Research. If there are other authors, they declare that they have no known competing financial interests or personal relationships that could have appeared to influence the work reported in this paper.

## Figures and Tables

**Table:** 

Target gene	NCBI accession	Forward primer sequence	Reverse primer sequence
*Rplp0*	NM_007475.5	AAA GAC TGG AGA CAA GGT GGG A	GTT GCG GAC ACC CTC CAG AA
*Gcg*	NM_008100.4	AGA CAG AAG CGC ATG AGG AC	TGG CAA TGT TGT TCC GGT TC
*Pcsk1*	NM_013628.3	GGT GGA AAG GTC GAG TCT AGC	TGC ACA CCA AAC GCA AAA GAA
*Pcsk2*	NM_008792.4	AAG AAG GAT GGA GGG CGG TT	CTG CAA AGG GGA GCT TTC GG
*Furin*	NM_011046.3	ATC CAC ATC TAC AGC GCC AG	CCT GGC TAA CTC CCC GAA AG
*Pcsk7*	NM_008794.2	GTC GAC ACT CAC TGC TCA CA	CAT CAG AAC AGC AGG CTG GG

**Table:** 

Reagent or resource	Source	Identifier
Antibodies		
Mouse anti-GLP1 [8G9]	AbCam	ab26278
Mouse anti-PC2 [EP3070]	R&D systems	MAB6018
Mouse anti-beta actin	ProteinTech	66009-1-Ig
Mouse anti-TGN38 [2F7.1]	Novus Biologicals	NB300-575
Mouse anti-calnexin [3H4A7]	Novus Biologicals	NBP1-47546
Rabbit anti-glucagon	AbCam	ab92517
Rabbit anti-PC1/3 [EPR21908]	AbCam	ab220363
Rabbit anti-Furin [E1W4O]	Cell Signaling Technology	70393T
Rabbit anti-PC7 [D4I5G]	Cell Signaling Technology	19346T
Guinea pig anti-insulin	DAKO/Agilent	A056401-2
Guinea pig anti-proglucagon	Progen Biotechnik	16032
Goat anti-Mouse AF568	ThermoFisher	A11004
Goat anti-Rabbit AF680	ThermoFisher	A21109
Goat anti-Guinea pig AF488	ThermoFisher	A11073
DAPI	Sigma	D9542-10 MG
Biological samples		
Human pancreatic islets	ADI IsletCore	R554, R561, R563
Human pancreatic islets	Clinical Islet Distribution Program	H2572, H2587, H2592, H2597, H2598, H2601
Chemicals, peptides, recombinant proteins		
Sitagliptin Phosphate	ApexBio	A4036-200
Recombinant human IL6	BioLegend	570804
Recombinant murine SDF-1α	ThermoFisher	25020A1UG
SC-79	Cayman Chemical	14972–5
Prolong Diamond Antifade	ThermoFisher	P36965
Accutase	StemPro	A1110501
Commercial Assays		
U-plex human GLP-1	MesoScale Discovery	K1515UK-1
U-plex human glucagon + insulin	MesoScale Discovery	K151ACM-1
Experimental models: Cell lines		
Mouse:αTC1/9 cells	ATCC	CRL-2350
Software and algorithms		
GraphPad Prism 10		
ImageJ		

## Data Availability

Data will be made available on request.

## Star methods

### Resource availability

Further information and requests for resources and reagents should be directed to the corresponding author (PEL, plight@ualberta.ca).

## Experimental model and sample details

### Cell culture

αTC1/9 cells from passage 38 to 48 were maintained at 11.1 mM glucose in a 37 °C incubator with 10% CO_2_. The complete culture medium contained low glucose DMEM supplemented with d-glucose to 11.1 mM, 5% fetal bovine serum, 0.02% bovine serum albumin, 15 mM HEPES, 100 μM non-essential amino acids, and 1% Penicillin/Streptomycin. Chronic incubation assays were conducted in complete media supplemented with 200 nM sitagliptin phosphate monohydrate and varying concentrations of cytokines. Cells were seeded in 24-well plates (∼10^5^ cells per well for RNA isolation and media collection) or 35 mm dishes (∼5 × 10^5^ cells per well for protein isolation), with media changes every 24 h.

### Human islet culture

Intact human islets (obtained from the Alberta Clinical Islet Transplant and Distribution Program [97], and Alberta Diabetes Institute IsletCore [90] in accordance with institutional human ethics guidelines) were maintained at 5.5 mM glucose in a 37 °C incubator with 10% CO2. The human islet culture medium contained low glucose DMEM, 5% fetal bovine serum, 100 mM sodium pyruvate, 15 mM HEPES, and 1% Penicillin/Streptomycin. Static hormone secretion assays were performed using HEPES-buffered Krebs–Ringer solution containing 140 mM NaCl, 3.6 mM KCl, 2.6 mM CaCl_2_, 0.5 mM NaH_2_PO_4_, 0.5 mM MgSO_4_, 5 mM HEPES, 2 mM NaHCO_3_, and 0.5 mg/mL Bovine Serum Albumin, with the addition of 200 nM sitagliptin phosphate monohydrate.

For single-cell assays, human islets were dispersed with accutase for 10–12 min at 37 °C, filtered through a 70 μm cell strainer, and maintained in 5.5 mM glucose complete human islet culture medium. All islet donor characteristics are summarized in Supplementary Table S1.

## Method details

### Quantitative PCR (qPCR)

Total RNA was extracted from αTC1/9 cells using Trizol (Invitrogen) following the manufacturer's protocol. RNA was eluted in ultrapure nuclease-free water and reverse transcribed using the iScript cDNA synthesis kit with 1 μg RNA per 10 μL reaction. qPCR assays were performed using Sybr Green reagent with 5 ng cDNA per reaction well on a Roche LightCycler480 thermocycler. Sample sizes ranged from 4 to 8 biological replicates run in technical duplicates. Ct values were determined using the absolute quantification second derivative maximum method. Sample purity and integrity were confirmed using the melt curve genotyping function in the Roche LightCycler 480 software (version 1.5.1.62 SP3).

### Primer design and validation

Primers were designed using the NCBI BLAST tool and synthesized by Sigma–Aldrich. Each primer set was validated using a standard curve of control cDNA (0 ng–10ng per reaction), with optimal concentrations titrated to 300 nM to prevent primer dimer formation. *Rplp0* was selected as a reference gene, with *Gcg* (proglucagon transcript) as a positive control for alpha cell identity. Primer pairs are summarized below.

### Cytokine incubation assays

αTC1/9 cells were seeded in 24-well plates or 35 mm dishes for 18–24 h in complete culture media before receiving experimental media conditions. Vehicle was complete αTC1/9 culture media at 5.5 mM glucose with 200 nM sitagliptin monophosphate, while experimental conditions contained either 1 ng/mL SDF1α, 2 ng/mL IL6, or 3 μM SC-79. Cells were maintained in vehicle or experimental conditions for 72 h, with media changes every 24 h. Media samples collected for downstream assays were briefly centrifuged, and the top fraction was transferred to fresh collection tubes and stored at −20 °C.

### Static hormone secretion assays

Human islets were handpicked to 99% purity and preincubated in HEPES-buffered Krebs Ringer solution for 3 h before allocation to assay tubes. 25 human islets were transferred to microfuge tubes and incubated in 5.5 mM glucose “basal” Krebs buffer for 1 h at 37 °C. Islets were briefly centrifuged, the supernatant was collected and placed at −20 °C, and media was replaced with “stimulating” condition Krebs solution. These stimulating conditions were HEPES-buffered Krebs Ringer buffer with either 2.5 mM glucose, 16.7 mM glucose, 5.5 mM glucose with 10 mM l-arginine, or 5.5 mM glucose with 10 mM l-alanine. Islets were incubated for 1 h at 37 °C before centrifugation and media collection. At the end of each assay, islets were fixed and lysed with acid ethanol solution (70% ethanol, 20% acetic acid, 1.5% HCl) before storage at −20 °C.

### Western blotting

αTC1/9 cells were lysed in buffer containing 50 mM Tris–HCl (pH 7.4), 1 mM EDTA, 1 mM EGTA, 1 mM Sodium Orthovanadate, 50 mM Sodium Fluoride, 5 mM sodium pyrophosphate, 250 mM Sucrose, 0.5% Triton-X 100, 1:100 protease inhibitor cocktail, and 1:50 phosphatase inhibitor cocktail. Lysates were centrifuged (4 °C, 200RCF, 5min) to remove debris. Protein concentrations were quantified by Bradford Assay and adjusted to 1 μg/μL in sample buffer containing 10% glycerol, 0.5% SDS, 0.025% bromophenol blue, and 62.5 mM Tris (pH 6.8). Proteins (40μg/lane) were separated on 10% SDS-PAGE and transferred to PVDF membranes using the PowerBlotter XL semi-dry transfer system (25V, 12min). Membranes were blocked for 1 h at room temperature before antibody incubation. Primary antibodies were incubated overnight at 4 °C, while secondary antibodies were incubated for 1 h at 22 °C protected from light. Alexa-Fluor conjugated secondary antibodies were detected using a BioRad ChemiDoc MP imaging system and appropriate fluorescence excitation/emission filter sets.

### Immunofluorescence and microscopy

αTC1/9 or dispersed human alpha cells were seeded on glass coverslips and fixed with 4% paraformaldehyde for 15 min at room temperature. Cells were permeabilized and blocked in 20% normal goat serum and 0.5% Tween-20 in PBS for 1 h. Primary antibodies were incubated for 1 h at 22 °C in buffer containing 5% goat serum and 0.5% Tween-20 in PBS. After washing, AlexaFluor-conjugated secondary antibodies were applied for 1 h at 22 °C in the dark. Cells were mounted with Prolong Diamond antifade reagent and imaged using an Olympus IX83 or Leica Stellaris 8 confocal microscope. Image analysis was performed using FIJI ImageJ (Version: 2.16/0/1.54p).

GLP-1(amide) antibody specificity was confirmed in our previous publication, where only GLP-1 1–36 amide, 7–36 amide and 9–36 amide were detected with no glucagon reactivity [3]. These findings are summarized in Supplementary Figure S4.

## Normalization, quantification, and statistical analysis

For ELISA assays, αTC1/9 cell GLP-1 content and secretion were normalized to 100 μg total protein as determined by Bradford protein assay. Human islet proglucagon-derived peptide concentrations were normalized to 1000μIU/mL insulin content from whole islet lysates. This normalization method was selected as islets from different donation sites had varying levels of entrapment.

All statistical analyses were conducted using GraphPad Prism 10. Data are presented as mean ± SEM. Statistical significance was set at P < 0.05. Power calculations were performed to ensure a minimum of 90% power with an alpha of 0.05 per assay type.
