# Supplementary material for: Furin may contribute to proglucagon processing and glucagon-like Peptide-1 production in human alpha cells
Source: Mol Metab. 2025 Sep 27;102:102259. doi: 10.1016/j.molmet.2025.102259 (PMC12547723; doi:10.1016/j.molmet.2025.102259)
Supplement: Multimedia component 1 [file mmc1.docx]

**Furin may contribute to proglucagon processing and Glucagon-Like Peptide-1 production in human alpha cells.**

Janyne Koepke, Wentong Long, Amy Barr, Peter E. Light*

Department of Pharmacology and the Alberta Diabetes Institute, Faculty of Medicine and Dentistry, University of Alberta, Edmonton, Alberta, Canada, T6G 2E1.

**Supplementary materials:**

| **Supplementary Table S1**: human islet donor characteristics and tissue usage | | | | | | | | | |
| --- | --- | --- | --- | --- | --- | --- | --- | --- | --- |
| Unique identifier | H2572 | H2587 | H2592 | H2597 | H2598 | H2601 | R554 | R561 | R563 |
| Age (years) | 28 | 15 | 60 | 27 | 22 | 42 | 23 | 54 | 59 |
| Donor Sex | M | M | M | M | M | M | F | F | M |
| BMI (kg/m2) | 24.2 | 27.7 | 32.1 | 27.9 | 23.1 | 22.6 | 30.3 | 27.3 | 26.6 |
| Donor HbA1C | 5.5 | 5.2 | 5.7 | 5.2 | 5.4 | 5.7 | 4.5 | 5.6 | 4.5 |
| HLA-A2 status | -ve | +ve | +ve | -ve | +ve | +ve | +ve | +ve | +ve |
| Isolation center | CIL AB | CIL AB | CIL AB | CIL AB | CIL AB | CIL AB | ADI IC | ADI IC | ADI IC |
| Diabetes history | No | No | No | No | No | No | No | No | No |
| Cause of death | NDD | DCD | NDD | NDD | NDD | NDD |  |  |  |
| Assays | histology | ELISA  WB | ELISA | ELISA | ELISA | histology | ELISA  histology | ELISA | ELISA |
| CIL AB – University of Alberta Clinical Islet Laboratory; ADI IC – Alberta Diabetes Institute IsletCore; NDD – neurological determination of death; DCD – donation after circulatory death | | | | | | | | | |


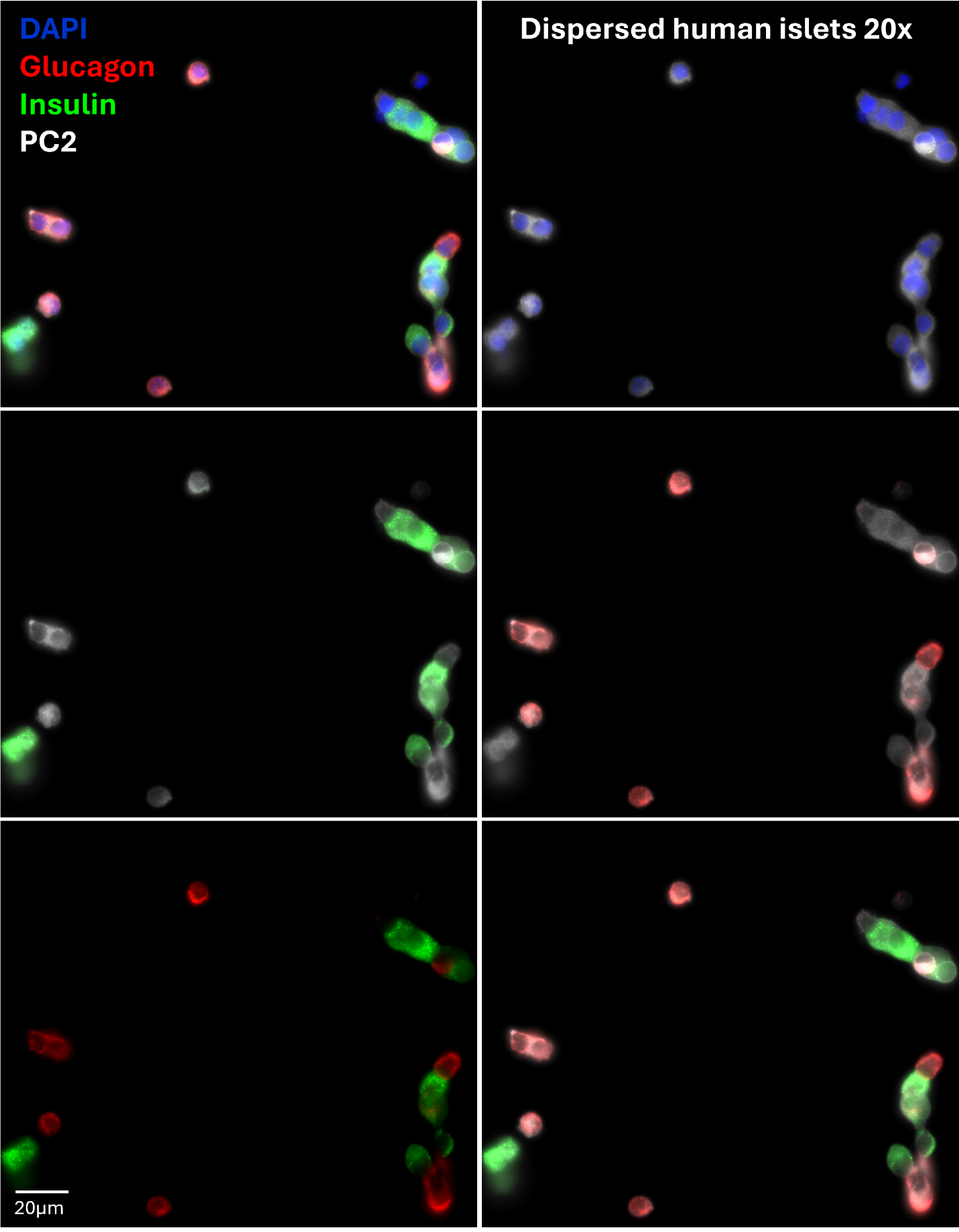


**Figure S1: Dispersed human islets planted on untreated glass coverslips; stained for glucagon, insulin, PC2**


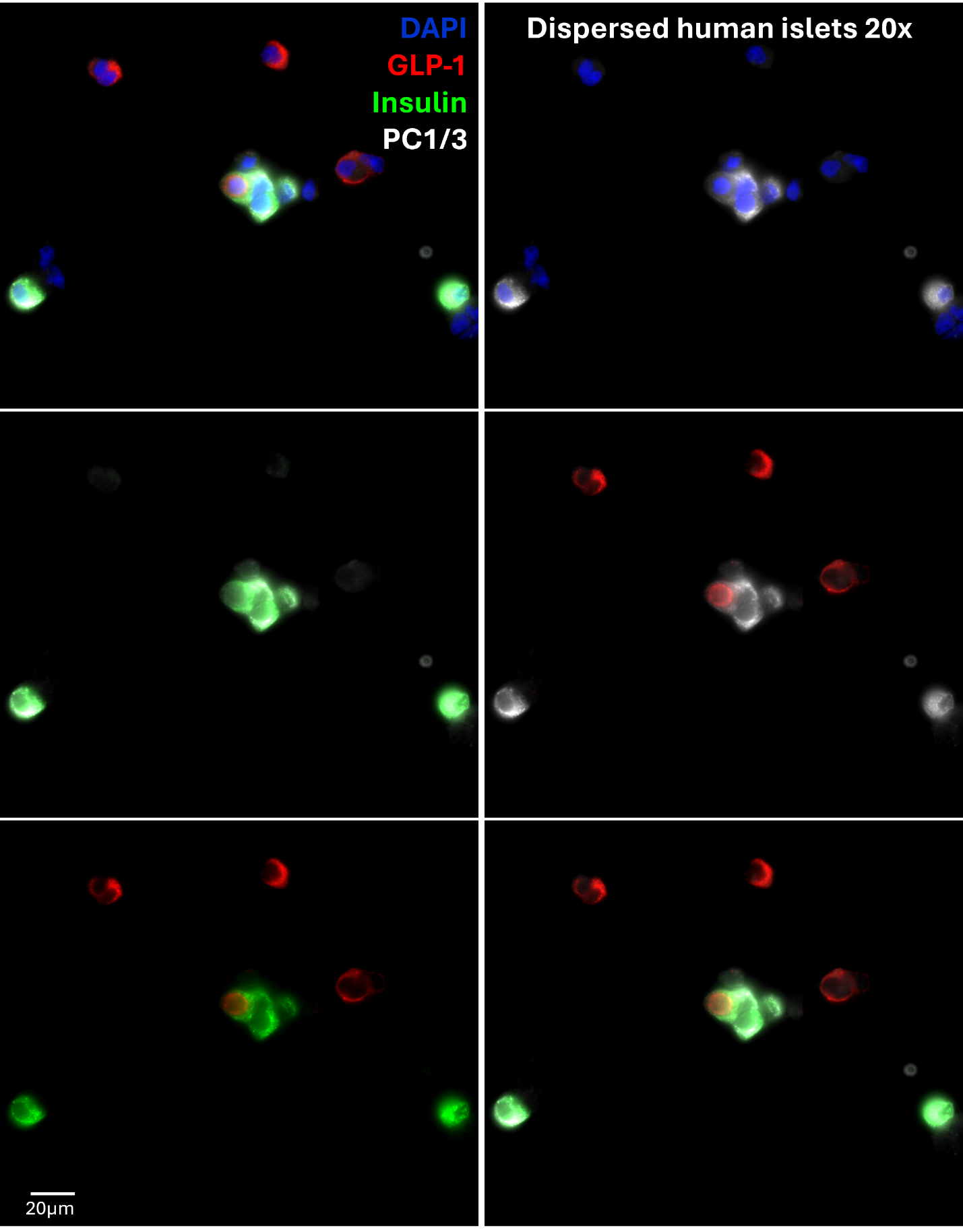


**Figure S2: Dispersed human islets planted on untreated glass coverslips; stained for GLP-1, insulin, PC1/3**


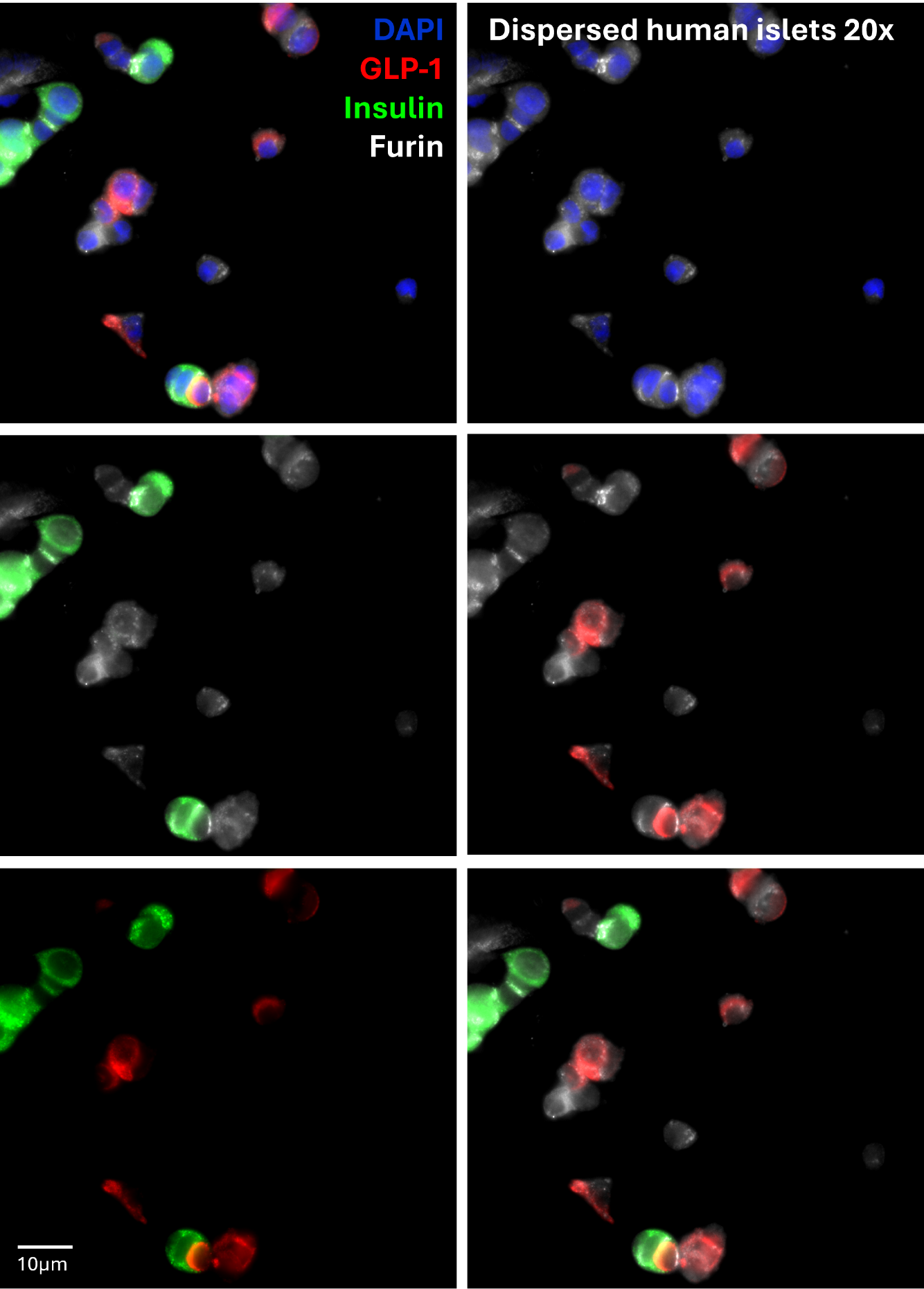


**Figure S3: Dispersed human islets planted on untreated glass coverslips; stained for GLP-1, insulin, furin**


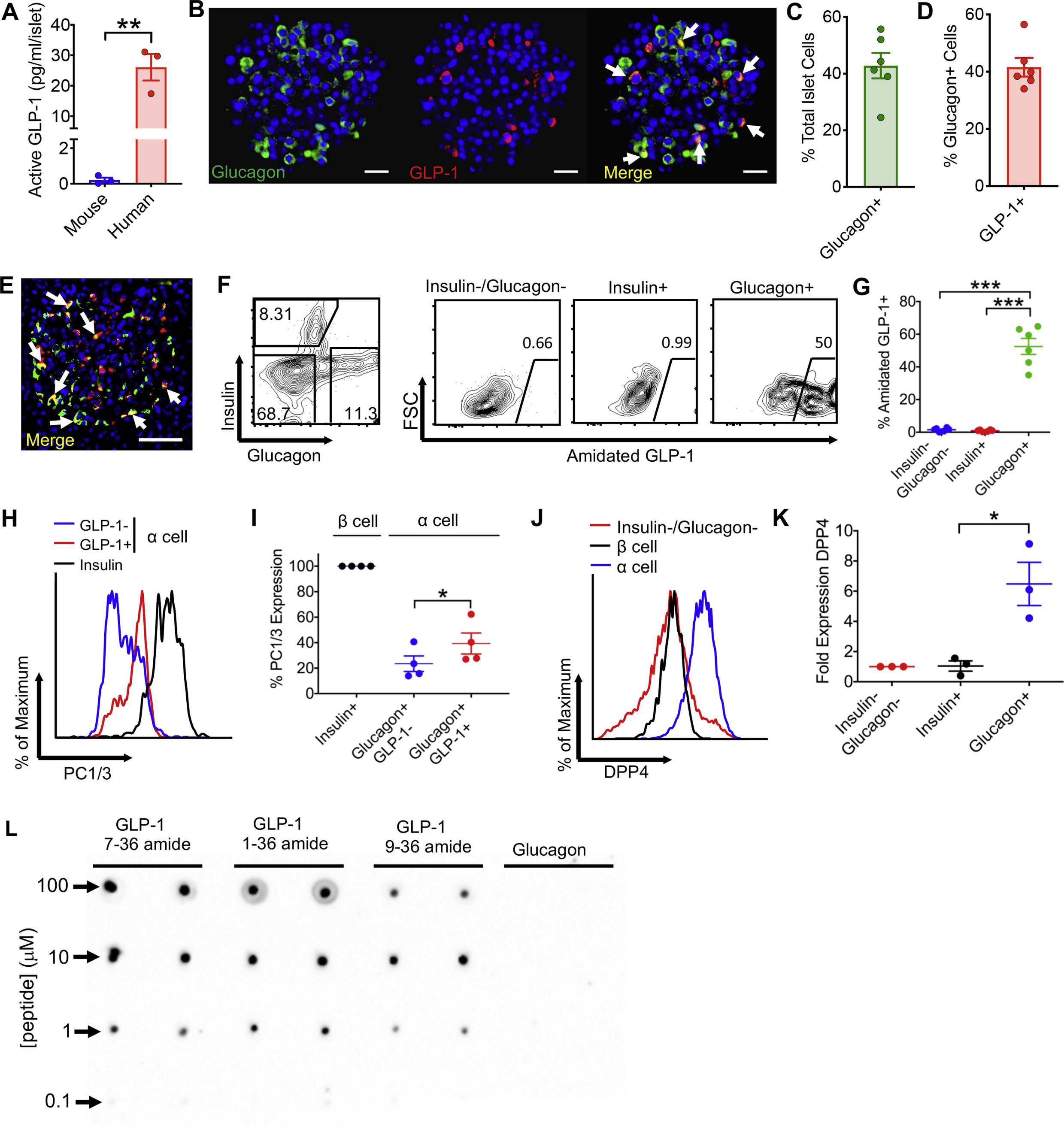


**Figure S4: Dot-blot of control peptides probed with GLP-1(amide) primary antibody ab26278. This antibody exhibits no glucagon reactivity.** Reproduced with permission from Campbell *et al.* 2020
